# Natural Products Derived from Marine Sponges with Antitumor Potential against Lung Cancer: A Systematic Review

**DOI:** 10.3390/md22030101

**Published:** 2024-02-23

**Authors:** Alba Ortigosa-Palomo, Francisco Quiñonero, Raul Ortiz, Francisco Sarabia, Jose Prados, Consolación Melguizo

**Affiliations:** 1 Institute of Biopathology and Regenerative Medicine (IBIMER), Biomedical Research Center (CIBM), 18100 Granada, Spain; albaortigosa@ugr.es (A.O.-P.); fjquinonero@ugr.es (F.Q.); roquesa@ugr.es (R.O.); melguizo@ugr.es (C.M.); 2 Instituto Biosanitario de Granada, (ibs.GRANADA), SAS-Universidad de Granada, 18012 Granada, Spain; 3 Department of Anatomy and Embryology, University of Granada, 18071 Granada, Spain; 4 Department of Organic Chemistry, Faculty of Sciences, University of Malaga, 29071 Malaga, Spain; frsarabia@uma.es

**Keywords:** marine sponge, bioactive compounds, antitumor, lung cancer

## Abstract

Non-small-cell lung cancer (NSCLC), the most commonly diagnosed cancer and the leading cause of cancer-related death worldwide, has been extensively investigated in the last decade in terms of developing new therapeutic options that increase patient survival. In this context, marine animals are a source of new, interesting bioactive molecules that have been applied to the treatment of different types of cancer. Many efforts have been made to search for new therapeutic strategies to improve the prognosis of lung cancer patients, including new bioactive compounds and cytotoxic drugs from marine sponges. Their antitumoral effect can be explained by several cellular and molecular mechanisms, such as modulation of the cell cycle or induction of apoptosis. Thus, this systematic review aims to summarize the bioactive compounds derived from marine sponges and the mechanisms by which they show antitumor effects against lung cancer, exploring their limitations and the challenges associated with their discovery. The search process was performed in three databases (PubMed, SCOPUS, and Web of Science), yielding a total of 105 articles identified in the last 10 years, and after a screening process, 33 articles were included in this systematic review. The results showed that these natural sponge-derived compounds are a valuable source of inspiration for the development of new drugs. However, more research in this field is needed for the translation of these novel compounds to the clinic.

## 1. Introduction

Lung cancer is one of the most common and lethal types of cancer worldwide. In 2020, 2.2 million new cases were diagnosed, resulting in 1.8 million deaths. These statistics position lung cancer as the cancer with the second-highest number of incidents and the leading cause of mortality, accounting for 11.4% of all cancer diagnoses and 18% of cancer-related deaths [1]. Based on histological characteristics, it can be categorized into two major types: non-small-cell lung cancer (NSCLC, comprising 85%) and small-cell lung cancer (SCLC, comprising 15%) [2]. NSCLC further divides into three subtypes: adenocarcinoma, squamous-cell carcinoma, and large-cell carcinoma, with the first two representing over 70% of NSCLC cases. Regarding the 5-year survival rate for lung cancer patients, it is less than 15%. This low survival rate is partly attributed to diagnoses in advanced stages, with over 60% of cases diagnosed in stages III or IV [3].

The therapeutic approach for stage I or II NSCLC entails surgical resection of the tumor, accompanied by adjuvant therapy. In contrast, as the disease progresses to stage III or IV, the therapeutic decision transitions toward chemotherapy or radiotherapy. Nevertheless, conventional chemotherapeutic agents exhibit some limitations, comprising low bioavailability, non-specific targeting, and the emergence of drug resistance [4]. Taken together, the development of innovative cancer treatments is crucial for improving survival rates, overcoming the adverse effects of current therapies, and mitigating challenges associated with tumor drug resistance [5].

Natural products have been used for a long time in the treatment of diseases, constituting a robust basis upon which modern pharmacology has been established and constitutes an accessible and interesting reservoir for the discovery and development of new bioactive molecules and new drugs. Roughly 80% of approved chemotherapeutic drugs and over half of all pharmaceuticals are derived from bioactive natural products [6]. In the context of cancer therapeutics, chemotherapeutic agents from natural origins have exerted a notable influence on the field. For instance, doxorubicin, paclitaxel, vincristine, and vinblastine are naturally derived compounds that are employed in cancer treatment as first-line drugs. Compounds sourced from marine environments have a more recent historical development [7].

The predominant portion of the Earth’s surface is comprised of water, constituting the seas and oceans, harboring an estimated one million unidentified species. These organisms represent a potentially valuable reservoir of bioactive secondary metabolites, serving as a source of inspiration for chemical synthesis aimed at enhancing their biological effects. Such endeavors hold promise for advancing the discovery and development of anticancer drugs [8]. The marine environment represents an enormous ecological reservoir housing a diverse array of aquatic plants and animals that possess significant antimicrobial, immunomodulatory, anti-inflammatory, anticancer, neuroprotective, analgesic, and antimalarial properties. Around 80% of the documented marine natural products (MNPs) belong to the phyla Porifera and Cnidaria, comprising 47.1% and 33.5%, respectively. The remainder is distributed among the phyla Echinodermata, Chordata, and Mollusca, accounting for 7.4%, 6%, and 5%, respectively [9]. The application of advanced technology and thorough investigation into marine natural products has yielded the identification of a novel class of anticancer drugs presently undergoing clinical trials. By 2022, there were seventeen marine-derived drugs approved for clinical use, 71% of which were used for the treatment of various types of cancer. This fact underscores the inherent antitumor efficacy of numerous molecules produced by marine organisms [10].

Sponges are sessile invertebrates that lack an innate immune system or physical defensive structures, such as spines or shells. Consequently, their primary mode of protection lies in the synthesis of secondary metabolites, serving as a self-defense mechanism that facilitates adaptation to their environment. Furthermore, the great biodiversity observed among sponge phyla contributes to the production of a diverse array of molecules with different chemical characteristics [9]. Approximately 30% of identified MNPs are sourced from sponges, exhibiting potential anticancer effects. These compounds encompass alkaloids, sterols, terpenoids, macrolides, polyketones, peptides, glycosides, and quinones [8]. The anticancer properties may be explained by the ability to regulate certain cellular and molecular processes, such as modulation of the cell cycle, apoptosis, and inflammation [9].

The aim of this systematic review is to provide an update on the potential antitumor effects against lung cancer of bioactive natural compounds derived from marine sponges, as well as chemical analogs, focusing on the mechanisms of action involved in their antiproliferative or cytotoxic effect. In addition, the limitations and challenges associated with the development of new drugs for the treatment of lung cancer will be critically examined.

## 2. Results

**Figure 1:**
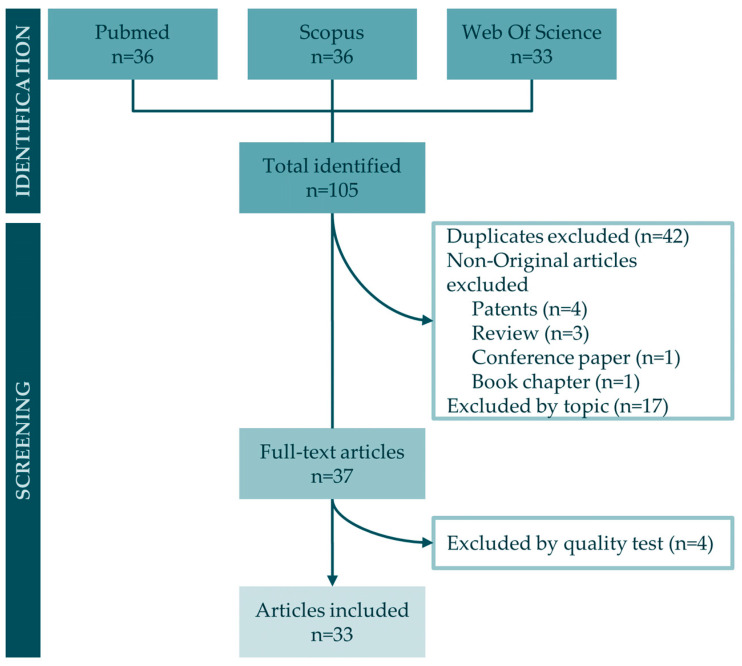
Workflow of articles included in this systematic review.

Following a literature search in the major online databases PubMed, Scopus, and Web of Science, a total of 105 articles were identified (36 from PubMed, 36 from Scopus, and 33 from Web of Science) based on the search equations described in the Materials and Methods section. Subsequent to the identification process, 42 duplicate publications were discerned and excluded from consideration. Furthermore, nine non-original articles, consisting of four patents, three reviews, one conference paper, and one book chapter, were omitted from the dataset. Additionally, 17 publications were excluded by topic and inclusion and exclusion criteria. Finally, a total of 37 full-text articles underwent rigorous quality tests, leading to the exclusion of 4 articles that failed to meet the predefined score threshold. As a result, the articles included in this systematic review comprise 33 publications. The workflow diagram of the search process is presented in Figure 1.

**Figure 2:**
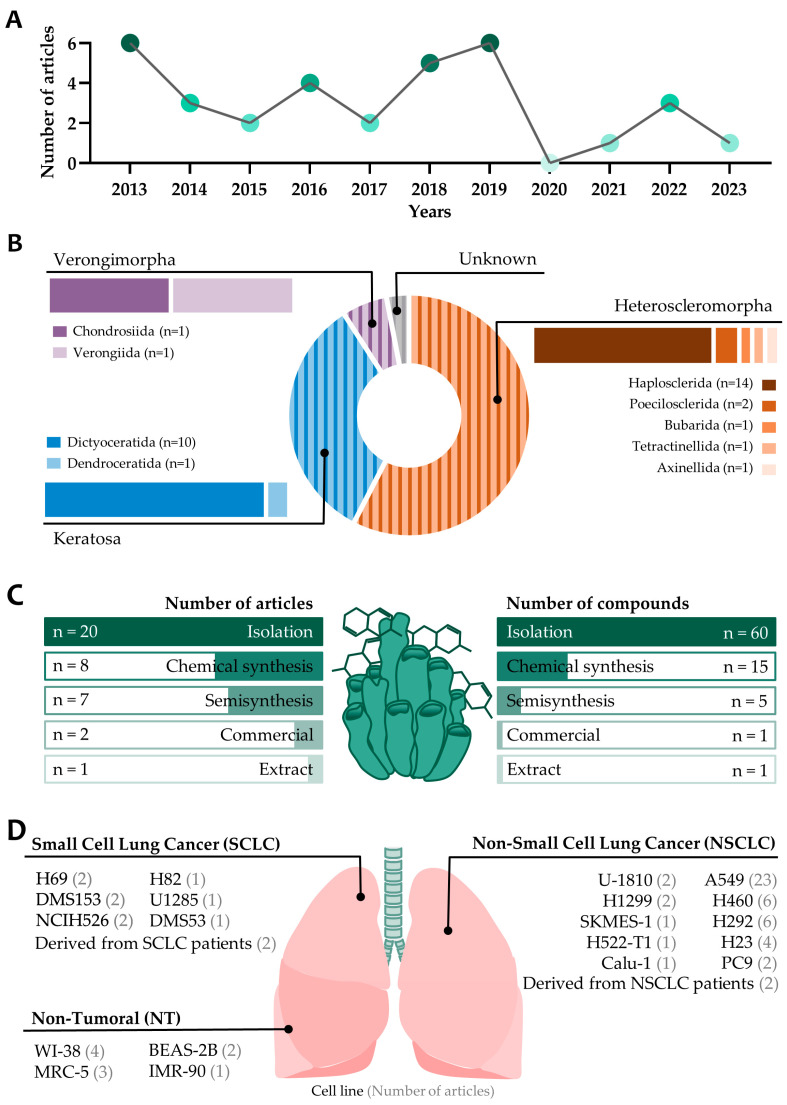
Graphical representation of the main characteristics of articles included in this systematic review. (**A**) Number of articles published per year (2013–2023) included for analysis in this systematic review. (**B**) Taxonomic categorization into subclass and order is performed for marine sponges serving as sources of compounds and extracts or as inspiration for synthesis. The number of articles is indicated in parentheses. (**C**) Graphical representation of the method for obtaining compounds included in this systematic review, quantifying the number of articles and compounds associated with each methodology. (**D**) Number of publications in which each cell line is used and its classification.

In terms of the number of articles published on the antitumor effects of compounds from marine sponges on lung cancer, no clear trend can be observed over the last ten years. Instead, 2013 and 2019 are the years in which the most papers were published (six articles) (Figure 2A). Of the 33 articles included in this systematic review, 19 of them included compounds isolated from the demosponge subclass Heterosclemorpha, 11 included compounds isolated from Keratosa, 2 included compounds isolated from Verongimorpha, and 1 article found a compound that was of unknown origin. Among them, the most common order is Haplosclerida (14 articles), followed by Dictyoceratida (10 articles) and Poecilosclerida (2 articles), leaving the rest of the orders, Bubarida, Tetractinellida, Axinellida, Dendroceratida, Chondrosiida, and Verongiida, with only one article including each of them (Figure 2B).

Regarding the method of obtaining the compounds used in the studies included in this systematic review (Figure 2C), most articles used isolation methods (20 publications), followed by chemically synthesized compounds (8 publications) and chemical modification from a natural compound obtained by isolation (7 publications). However, if the focus shifts from analyzing the methodologies employed in the publications to examining the number of compounds obtained through each pathway, it is determined that out of a total of 82 compounds, 60 were acquired through isolation, 15 through semisynthesis, 5 through chemical synthesis, 1 was procured from a commercial source, and 1 was an extract. The methods of isolation are indispensable for the discovery of novel molecules exhibiting antitumoral activity. Nevertheless, chemical synthesis provides a highly advantageous tool as it allows for the modification of natural molecules to enhance their properties. Moreover, it inspires researchers to generate chemical analogs, from which groups of compounds with slight structural modifications can be produced, enabling the identification of the most potent candidate for further analysis. Chemical modification can also mitigate the undesired effects of the original molecule, as demonstrated in the work conducted by Cheun-Arom et al. (2013), wherein the chemical modification of renieramicyn M reduces the necrotic effects induced by the natural compound [11].

Based on histological characteristics, lung cancer is categorized into non-small-cell lung cancer (NSCLC) and small-cell lung cancer (SCLC), with the former being more prevalent and the latter more lethal. In Figure 2D, cell lines corresponding to each histological type of lung cancer and non-tumoral cell lines are represented, along with the number of articles included in this systematic review in which they are used. The three most extensively studied cell lines were of NSCLC, with the A549 cell line being the most commonly employed, specifically in 23 out of 33 articles, followed by the H460 and H292 cell lines.

### 2.1. Order Heterosclemorpha

**Figure 3:**
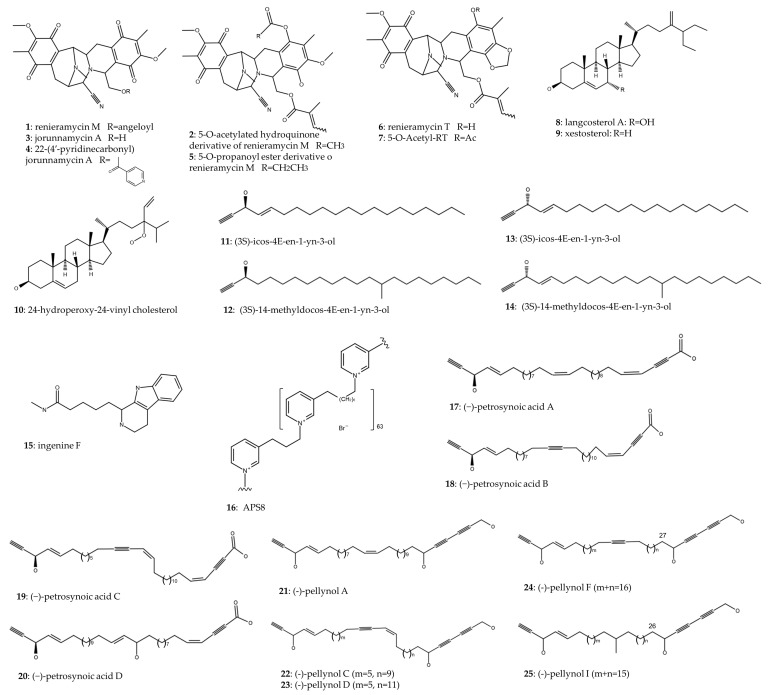
Chemical structure of compounds from Haplosclerida order.

Table 1 shows marine sponges of the order Heterosclemorpha and compounds synthesized or isolated (Figure 3) from them with antitumor activity against lung cancer. Eight of the fourteen articles included in this table feature marine sponges of the genus *Xestospongia*. Renieramycin M (**1**) has been studied in three articles. Cheun-Arom et al. (2013) reported that renieramycin M (**1**) isolated from *Xestospongia* sp. had an accidental necrosis-inducing effect, which limits its development by unwanted toxicity. Therefore, by chemical modification of renieramycin M (**1**), they synthesized a 5-O-acetylated hydroquinone derivative (**2**), which maintained the cytotoxic effect (IC_50_ ~1 μM in H23 cell line) and apoptosis levels while reducing the generation of ROS, which is responsible for necrosis [11]. Furthermore, Sirimangkalakitti et al. (2016) prepared eighteen 22-O-ester derivatives of jorunnamycin A (**4**) isolated from *Xestospongia* and tested their cytotoxic effect on two NSCLC lines. The most potent compound, the 22-O-(4-pyridinecarbonyl) ester derivative of jorunnamycin A (**3**), showed a 21- and 5-fold IC_50_ decrease in H292 and H460 NSCLC cell lines, respectively, compared to renieramycin M (**1**) [12]. On the other hand, similarly, Chamni et al. (2017) performed a chemical synthesis of several hydroquinone 5-O-monoesteranalogs of renieramycin M, and the best result was observed in the H292 cell line, in which a 5-O-propanoyl ester derivative (**5**) managed to reduce the IC_50_ (2.3 nM) 10-fold with respect to renieramycin M (24 nM) [13].

Another renieramycin, renieramycin T (**6**), was isolated from Thai blue sponge *Xestospongia* sp. and was shown to be predominantly toxic in NSCLC cells compared to the non-tumor cell line BEAS-2B. The cytotoxic effect was exerted through the induction of apoptosis. Renieramycin T (**6**) caused the activation of p53 and caspases 9 and 3, while it promoted the degradation of Mcl-1, a proapoptotic protein belonging to the Bcl-2 family, in the proteasome [14]. 5-O-acetyl-renieramycin T (**7**), chemically synthesized from renieramycin T (**6**) isolated from *Xestospongia*, showed a cytotoxic effect on H292, A549, and H23 with IC_50_ of 0.66, 33.24 and 33.77 μM, respectively. They continued studying the mechanisms of action on H292 and reported cell death by p-53 activation-dependent apoptosis, as well as a decrease in the cancer stem cell (CSC) population and increased sensitivity of H292 cells to cisplatin [15].

22-(4′-pyridinecarbonyl) jorunnamycin A (22-(4′-py)-JA) (**4**) is a synthetic derivative of jorunnamycin A (**3**) isolated from *Xestospongia* sp. [16,17]. This compound demonstrated robust cytotoxic efficacy in the nanomolar range, exerting an apoptosis-inducing effect on H460, H292, and A549 NSCLC cell lines through an ERK/MEK/Bcl-2-dependent mechanism [16]. It has also been shown to reduce cell invasion in NSCLC and angiogenesis in HUVECs. In vitro assays demonstrated that inhibition of AKT/mTOR/P70S6K signaling pathway by 22-(4′-py)-JA resulted in the attenuation of matrix metalloproteinases (MMP-2 and MMP-9), hypoxia-inducible factor-1α (HIF-1α), and vascular endothelial growth factor (VEGF). In vivo assays involving intravenous inoculation of the A549 cell line in mice revealed that the group treated with 22-(4′-py)-JA (**4**) exhibited a reduction in pulmonary nodules, thereby mitigating the metastatic progression [17]. Cell invasion and angiogenesis processes are related to the spread of tumor cells, leading to metastasis. In this regard, this compound was associated with downregulation of MMP-2 and MMP-9, related to cell invasion [18], and HIF-1α and VEGF, related to angiogenesis [19].

Nguyen et al. (2019) successfully isolated three sterols from the marine sponge *Xestospongia testudinaria*. Among these, langcosterol A (**8**) and 24-hydroperoxy-24-vinyl cholesterol (**10**) exhibited cytotoxic activity against the A549 cell line with IC_50_ of 63 and 29 μM, respectively. Moreover, their cytotoxic effect was assessed in a non-tumor cell line, WI-38, revealing an IC_50_ increase of 1.1 and 1.5 times in comparison to the IC_50_ values observed in the A549 cell line [20].

Two of the fourteen articles included in Table 2 highlight marine sponges belonging to the genus *Cribrochalina*. Zovko et al. (2014) successfully isolated two acetylenic compounds from *Cribrochalina vasculum*, (3S)-icos-4E-en-1-yn-3-ol (**11**) and (3S)-14-methyldocos-4E-en-1-yn-3-ol (**12**), demonstrating significant tumor-specific toxicity in NSCLC cell line U-1810 and SCLC cell lines U-1285, H69, and H82. Conversely, these compounds exhibited a less pronounced effect on non-tumor cell lines WI-38 and BEAS-2B. Action mechanism studies conducted in the U-1810 cell line revealed that the compounds induced apoptosis through the cleavage of caspase-9, caspase-3, and PARP. Subsequent analyses demonstrated conformational alterations in Bak and Bax, leading to mitochondrial potential loss and cytochrome C release. Additionally, the compounds were associated with decreased phosphorylation of Akt, mTOR, and ERK, along with increased phosphorylation of JNK [21]. Other researchers isolated two distinct acetylenic compounds, (3R)-icos-(4E)-en-1-yn-3-ol (**13**) and (3R)-14-methyldocos-(4E)-en-1-yn-3-ol) (**14**), and demonstrated their cytotoxic effects in the U-1810 cell line. The elucidation of their mechanism of action revealed a reduction in the phosphorylation of IGF-1R, thereby inhibiting pro-survival signaling pathways [22].

Hadisaputri et al. (2021) prepared a methanolic extract from the marine sponge *Callyspongia aerizusa*, exhibiting robust cytotoxicity and colony inhibition capability in the A549 cell line (IC_50_ = 9.38 μg/mL). The extract induced the upregulation of proapoptotic proteins, including caspase-3, caspase-9, and PARP-1, while concurrently inhibiting the antiapoptotic protein Bcl-2 [23]. Ingenine F (**15**), a 1,2,3,4-tetrahydro-β-carboline (THβC) alkaloid isolated from *Acanthostrogylophora ingens*, showed an IC_50_ value of 2.37 μM toward A549 cell line [24].

Zovko et al. (2013) synthesized a chemical analog, APS8 (**16**), inspired by the 3-alkylpyridinium polymer found in *Reniera sarai*. The study demonstrated that APS8 significantly reduced cell viability in A549 (IC_50_ = 375 nM) and SKMES-1 (IC_50_ = 362 nM) cell lines. Conversely, in the non-tumor MRC-5 cell line, a concentration of 1 μM led to only a 20% reduction in viability. APS8 induced apoptosis and mitochondrial membrane depolarization, specifically in NSCLC cells but not in non-tumor cells. Furthermore, APS8 increased the expression of proapoptotic proteins while decreasing the expression of antiapoptotic proteins. Notably, it also increased caspase-9 activation, specifically in tumor cell lines [25]. In their study, Mejia et al. (2013) extracted nine compounds, namely the (-)-petrosynoic acids A-D (**17**–**20**) and five pellynols, A (**21**), C (**22**), D (**23**), F (**24**), and I (**25**), from the marine sponge *Petrosia* (order Haplosclerida). Surprisingly, while these compounds exhibited a potent cytotoxic effect on NSCLC cell lines, the non-tumor cell line IMR-90 displayed lower IC_50_ values for almost all compounds compared to the tumor cell lines [26].

Among all the compounds belonging to Haplosclerida order, renieramycin M (**1**) and the derivatives of jorunnamycin A (**4**) and hydroquinone 5-O-monoester (**2**) stand out for their high antitumor activity against NSCLC lines H292 and H460, obtaining the lowest IC_50_ [11,12]. In addition, the derivatives of compound **4** have been the only ones within this order that have been tested in in vivo assays with mice, obtaining interesting antimetastatic effects through the deregulation of the expression of metalloproteases and signaling pathways of this phenomenon (HIF and VEGF) [17]. Notably, they all belong to the alkaloid family. Different types of alkaloids have been previously characterized as potent tumor suppressors through the alteration of several cell signaling pathways involved in cell division, cell cycle progression, and metastasis [27]. Most of these antitumor compounds have been isolated from plants, and some of them, such as coclaurine and reflexin A, among others, have shown interesting antitumor effects. However, a major disadvantage of these compounds is their low solubility and bioavailability, which makes their administration as free molecules complicated [28].

**Table 1:** Antitumor activity of compounds from Haplosclerida order in lung cancer.

Sponge	Compounds	Chemical Class	Methods of Production	Cell line (NSCLC/SCLC)	IC50	In Vivo *	Molecular Mechanism	Ref.
*Xestospongia* sp.	renieramycin M (**1**) and 5-O-acetylated hydroquinone derivative (**2**)	1: Bistetrahydroisoquinolinequinone alkaloid 2: Hydroquinone derivative	**1**: Isolation **2**: Semisynthesis. Chemical modification of (**1**)	H23 (NSCLC)	**1** and **2**: ~1 μM (24 h)	✗	(**2**) reduced the accidental necrosis-inducing effect while preserving the apoptosis-inducing effect of (**1**).	[11]
*Xestospongia* sp.	renieramycin M (**1**), jorunnamycin A (**3**), and 22-O-(4-pyridinecarbonyl) ester derivative of jorunnamycin A (**4**)	Alkaloid	**1** and **3**: Isolation **4**: Semisynthesis. Chemical modification of (**2**)	H292 and H460 (NSCLC)	**1**: 23 ± 4 and 8.3 ± 0.6 **3**: 220 ± 20 and 160 ± 10 **4**: 1.1 ± 0.1 and 1.6 ± 0.3 IC_50_ H292 and H460 (nM), respectively (72 h)	✗	NR	[12]
*Xestospongia* sp.	renieramycin M (**1**), 5-O-acetyl ester (**2**), and 5-O-propanoyl ester (**5**) derivatives	Alkaloid	**1**: Isolation **2** and **5**: Semisynthesis. Chemical modification of (**2**)	H292 and H460 (NSCLC)	**1**: 24 ± 1 and 6.5 ± 0.4 **2**: 3 ± 0.6 and 3.6 ± 0.6 **5**: 2.3 ± 0.4 and 5.1 ± 0.5 IC_50_ H292 and H460 (nM), respectively (72 h)	✗	NR	[13]
*Xestospongia* sp.	renieramycin T (**6**)	Tetrahydroisoquinoline alkaloid	Isolated	H460, H292, H23, A549 (NSCLC), and BEAS-2B (NT)	1.93 ± 0.4, 0.88 ± 0.06, 2.47 ± 0.14, 3.77 ± 0.38, 6.42 ± 0.65 IC_50_ H460, H292, H23, A549, and BEAS-2B (μM), respectively (24 h)	✗	Apoptosis induction through Mcl-1 (antiapoptotic protein), proteasomal degradation, and activation of p53, caspase-9 and -3, and PARP.	[14]
*Xestospongia* sp.	5-O-acetyl-renieramycin T (**7**)	Alkaloid	Semisynthesis. Chemical modification of renieramycin T	H292, A549, and H23 (NSCLC)	0.66 ± 0.26, 33.24 ± 4.75, 33.77 ± 2.22 IC_50_ H292, A549, and H23 (μM), respectively	✗	Induction of p53-dependent apoptosis, suppressing expression of CSC markers and depleting AKT signal.	[15]
*Xestospongia* sp.	22-(4′-pyridinecarbonyl) jorunnamycin A (22-(4′-py)-JA) (**4**)	Tetrahydroisoquinoline derivative	Semisynthesis. Chemical modification of JA	A549, H460, and H292 (NSCLC)	14.43 ± 0.68, 18.9 ± 0.76, 16.95 ± 0.41 IC_50_ A549, H460, and H292 (nM), respectively (48 h)	✗	Induction of apoptosis in an ERK/MEK/Bcl-2-dependent manner.	[16]
*Xestospongia* sp.	22-(4′-pyridinecarbonyl) jorunnamycin A (22-(4′-py)-JA) (**4**)	Tetrahydroisoquinoline derivative	Semisynthesis. Chemical modification of JA	A549 and H460 (NSCLC)	810 ± 33, 19 ± 1, and 11 ± 0.5 IC50 A549 (nM) at 24, 48, and 72 h, respectively 835 ± 30, 14 ± 1, and 12 ± 0.4 IC_50_ H460 (nM) at 24, 48, and 72 h, respectively	✓	Suppression of AKT/mTOR/p70S6K signaling, leading to the downregulation of MMP-2 and MMP-9, HIF-1α, and VEGF. Reduction of in vivo metastasis.	[17]
*Xestospongia testudinaria*	langcosterol A (**8**), xestosterol (**9**), and 24-hydroperoxy-24-vinyl cholesterol (**10**)	Sterol	Isolation	A549 (NSCLC) and WI-38 (NT)	**8**: 63.1 and 68 **9**: >100 and >100 **10**: 29 and 43.4 IC_50_ (μM) A549 and WI-38, respectively (72 h)	✗	NR	[20]
*Cribrochalina vasculum*	(3S)-icos-4E-en-1-yn-3-ol (**11**) and (3S)-14-methyldocos-4E-en-1-yn-3-ol (**12**)	Acetylenic alcohol	Isolation	U-1810 (NSCLC), U-1285, H69, H82 (SCLC), WI-38, and BEAS-2B (NT)	**11**: 0.5, 1.6, 2.2, 1.1, 7.3, and 12.9 **12**: 0.8, 1.8, 1.1, 1.1, 9.7, and N/A IC_50_ (μM) U-1810, U-1285, H69, H82, WI-38, and BEAS-2B, respectively (72 h)	✗	Induction of apoptosis involving cleavage of caspase-9, caspase3, and PARP. Conformational changes in Bak and Bax and loss of mitochondrial potential and cytochrome release. Decreased phosphorylation of Akt, mTOR, and ERK and increased phosphorylation of JNK. Cell cycle arrest in G_2_/M.	[21]
*Cribrochalina vasculum*	(3R)-icos-(4E)-en-1-yn-3-ol (**13**) and (3R)-14-methyldocos-(4E)-en-1-yn-3-ol) (**14**)	Acetylenic alcohol	Isolation	U-1810 (NSCLC)	**13**: 1.5 μM **14**: 15.1 μM (72 h)	✗	(**13**) Inhibition of IGF-1R phosphorylation, thus reducing pro-survival signaling.	[22]
*Callyspongia aerizusa*	methanol extraction	Ergosteroid (suggested to suppress cell viability)	Extract	A549 (NSCLC)	9.38 μg/mL (24 h)	✗	Induction of apoptosis. Increase in PARP-1 and caspase-3 and -9 and decrease in Bcl-2.	[23]
*Acanthostrongylophora ingens*	ingenine F (**15**)	1,2,3,4-tetrahydro-β-carboline (THβCs) alkaloid	Isolation	A549 (NSCLC)	2.37 μM	✗	NR	[24]
*Reniera* sarai renamed to *Haliclona (Rhizoneira) sarai*	analog of 3-alkylpyridinium polymer (APS8) (**16**)	Polymeric alkylpyridinium salt	Chemical synthesis	A549 and SKMES-1 (NSCLC) and MRC-5 (NT)	375 ± 4.89, 362 ± 9.29, and >1000 IC_50_ (nM) A549, SKMES-1, and MRC-5, respectively (48 h)	✗	Apoptosis, mitochondrial membrane depolarization, upregulation of several proapoptotic proteins, downregulation of antiapoptotic proteins, and activation of caspase-9.	[25]
*Petrosia* sp.	petrosynoic acid A (**17**), petrosynoic acid B (**18**), petrosynoic acid C (**19**), petrosynoic acid D (**20**), pellynol A (**21**), pellynol C (**22**), pellynol D (**23**), pellynol F (**24**), and pellynol I (**25**)	**17**–**20**: (−)-petrosynoic acids **21**–**25**: pellynols	Isolation	H522-T1 and H460 (NSCLC) and IMR-90 (NT)	**17**: 5.5, 3.4, and 0.4 **18**: 8.4, 7.3, and 0.4 **19**: 4.6, 3.1, and 0.3 **20**: >10, >10, and 0.2 **21**: 0.8, 0.4, and 0.4 **22**: 0.6, 1, and 0.3 **23**: 1.1, 0.7, and 0.5 **24**: 2.7, 1.2, and 1.6 **25**: 1.2, 0.8, and N/A IC_50_ (μM) H522-T1, A549, and IMR-90 (72 h)	✗	NR	[26]

* in vivo in lung cancer; not reported, NR; non-small-cell lung cancer, NSCLC; small-cell lung cancer, SCLC; non-tumoral, NT; induced myeloid leukemia cell-differentiation protein Mcl, Mcl-1; tumor protein P53, p53; cancer stem cell, CSC; protein kinase B, Akt; extracellular signal-regulated kinase, ERK; mitogen-activated protein kinase, MEK; B-cell lymphoma 2, Bcl-2; serine/threonine-protein kinase, mTOR; ribosomal protein S6 kinase B1, p70S6K; matrix metalloproteinase, MMP; hypoxia-inducible factor 1-alpha, HIF-1α; vascular endothelial growth factor A, VEGFA; poly (ADP-ribose) polymerase, PARP; Bcl-2 homologous antagonist/killer, Bak; BCL2-associated X, Bax; insulin-like growth factor 1 receptor, IGF-R1; no in vivo evaluation in lung cancer, ✗; in vivo evaluation in lung cancer, ✓.

### 2.2. Order Dictyoceratida

**Figure 4:**
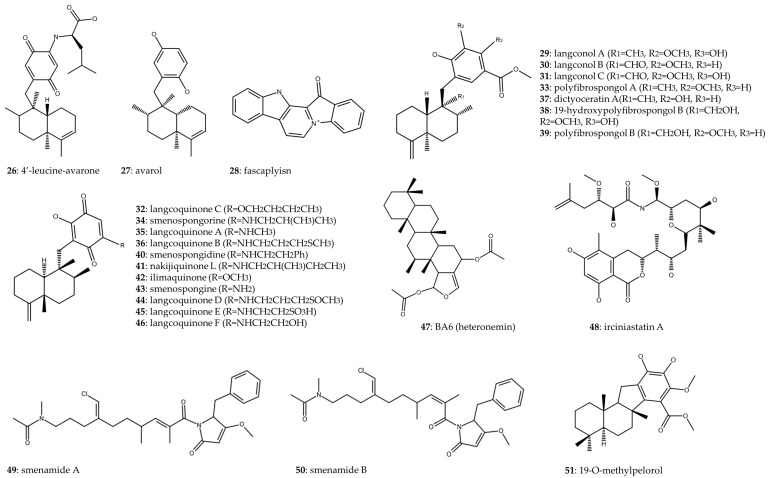
Chemical structure of compounds from Dictyoceratida order.

Table 2 presents marine sponges belonging to the order Dictyoceratida along with the compounds (Figure 4) either synthesized or isolated from them, demonstrating their antitumor activity against lung cancer. Notably, out of the fourteen articles incorporated into this table, two highlight marine sponges from the genus *Dysidea*. Avarol (**27**), isolated from *Dysidea avara*, was examined for its antiproliferative effects on A549 and MRC-5, demonstrating IC_50_ values of 35.27 and 29.14 μg/mL, respectively. In vivo assays using murine models of Ehrlich carcinoma and cervical cancer revealed a significant 29% and 36% reduction in tumor size, respectively, compared to the control group [29]. In a related study, 4′-leucine-avarone (**26**) was synthesized from avarol (**27**) obtained from the same marine sponge species. Notably, this compound exhibited increased specificity toward the A549 cell line (IC_50_ = 7.4 μM) compared to the non-tumorous MRC-5 cell line (IC_50_ > 100 μM) [30].

Two scientific articles investigate the compound fascaplysin (**28**), which is naturally found in the marine sponge *Fascaplysinopsis Bergquist*, and focus their research on SCLC. In the study conducted by Hamilton (2014), the cytotoxic impact of fascaplysin (**28**) was assessed on NSCLC, SCLC, and various cell lines derived from SCLC patients with IC_50_ ranging from 134 to 1740 nM. Fascaplysin (**28**) induced cell cycle arrest at G_1_/G_0_ at lower concentrations and at S phase at higher concentrations, generated reactive oxygen species (ROS), and triggered cell death, particularly in the chemoresistant NCI-H417 cell line [31]. The generation of these ROS was previously associated with damage to nucleic acids, proteins, lipids, organelles, and membranes, leading to cell death [32]. Otherwise, the regulation of the cell cycle is a key factor in maintaining cellular homeostasis. In this case, fascaplysin (**28**) produced S phase arrest, preventing DNA replication and thus inhibiting tumor cell proliferation. These cell cycle arrest effects have also been observed with extracts and compounds derived from other marine sponges in in vitro assays in Burkitt’s lymphoma [33], colorectal cancer [34], breast cancer [35], and melanoma [36]. Rath et al. (2018) also studied the cytotoxic effect of fascaplysin (**28**) on cell lines from NSCLC and SCLC, cells derived from pleural effusions of NSCLC and SCLC patients, and circulating tumor cells (CTCs) from SCLC patients. The IC_50_ values ranged from 0.2 to 1.48 μM for SCLC and from 0.63 to 2.04 μM for NSCLC. Fascaplysin (**28**) exhibited cytotoxic activity against CTCs, which are associated with poor disease prognosis and metastatic development. Additionally, the study revealed the activation of an ATM-triggered signaling cascade in response to drug-induced DNA damage [37].

Fifteen merosquiterpenes (**29**–**43**) were isolated from the marine sponge *Spongia* sp., and their antiproliferative activity against A549 and the non-tumor cell line WI-38 was demonstrated, with respective IC_50_ values provided in Table 2 [38]. Additionally, in a separate study, three sesquiterpenoid quinones (**44**–**46**) were isolated from the same sponge. Among these compounds, langcoquinone D (**44**) exhibited the most potent cytotoxic effects, demonstrating IC_50_ values of 8.9 μM for A549 and 5.6 μM for WI-38, thus showing no specificity toward tumor cell lines [39].

Cheng et al. (2019) isolated the sesterterpene BA6 (heteronemin) (**47**) from the marine sponge *Hyrtios erecta* and determined its IC_50_ to be 5.12 μM in A549 cells. BA6 (**47**) exhibited a mechanism of action involving an increase in mitochondrial reactive oxygen species (mtROS) production, resulting in mitochondrial dysfunction. Furthermore, BA6 (**47**) upregulated the proapoptotic protein Bax, downregulated the antiapoptotic protein Bcl-2, promoted the release of cytochrome C, and activated caspase-9 and -3, ultimately inducing apoptosis in lung cancer cells [40]. Apoptosis is a widely studied mechanism seen in several of the compounds mentioned above. In fact, some of the commercial drugs used to treat lung cancer, such as paclitaxel and cisplatin, have been shown to promote apoptosis, being one of the main pathways of death induction in tumor cells [41,42].

Irciniastatin A (**48**), also recognized as psymberin, is a marine compound initially discovered in the marine sponges *Ircinia ramose* and *Psammocinia*. Quach et al. (2015) successfully synthesized this compound through chemical synthesis. Although they did not quantify the cytotoxic effect of irciniastatin A (**48**), their study focused on its mechanism of action on A549 cells. Irciniastatin A (**48**) was identified as a translation inhibitor, triggering activation of ERK, p38 MAP kinase, and JNK pathways. Consequently, this activation led to the shedding of the ectodomain of TNF receptor 1 [43]. Two compounds, namely smenamide A (**49**) and B (**50**), were isolated from *Smenospongia aurea* and investigated for their cytotoxic impact on the Calu-1 cell line. Both compounds demonstrated closely comparable IC_50_, with values of 48 and 49 nM, respectively. Additionally, their cytotoxic effects were mediated through the induction of apoptosis [44]. In a study conducted by J. Li et al. (2018), 14 meroterpenoids were isolated from the marine sponge *Dactylospongia* sp., and their anti-inflammatory and cytotoxic properties were investigated. Among the compounds examined, only 19-O-methylpelorol (**51**) demonstrated cytotoxicity against the PC-9 cell line, with IC_50_ of 9.2 μM [45].

Among the compounds derived from Dictyoceratida order, the most interesting compounds for their high cytotoxic activity were smenamide A (**49**) and smenamide B (**50**), with IC_50_ values of 48 and 49 nM, respectively, in the NSCLC cell line Calu-1 [44]. Both compounds belong to the peptide/polyketide hybrid family, generated by polyketides assembled through a non-ribosomal peptide synthetase in an RNA-independent pathway [46]. It should be noted that the behavior is maintained among different members of this family, having found that other similar compounds, such as smenothiazole A and B, also showed a high antitumor capacity, with IC_50_ values in the nM range in (CALU-1 and LC31) lung cancer cell lines. These compounds also generated cell death through the induction of the apoptotic pathway and by inducing a cycle arrest in the G_0_/G_1_ phase, which implies an inability to progress in cell proliferation [47].

**Table 2:** Antitumor activity of compounds from Dictyoceratida order in lung cancer.

Sponge	Compounds	Chemical Class	Methods of Production	Cell Line (NSCLC/SCLC)	IC50	In Vivo *	Molecular Mechanism	Ref.
*Dysidea avara*	4′-leucine-avarone (**26**)	Amino derivative	Semisynthesis. Chemical modification of avarol	A549 (NSCLC) and MRC-5 (NT)	7.40 ± 2.98 and >100 IC_50_ (μM) A549 and MRC-5, respectively (48 h)	✗	NR	[30]
*Dysidea avara*	avarol (**27**)	Sesquiterpene hydroquinone	Isolation	A549 (NSCLC) and MRC-5 (NT)	35.27 ± 0.64 and 29.14 ± 0.41 IC_50_ (μg/mL) A549 and MRC-5, respectively (72 h)	✗	NR	[29]
*Fascaplysinopsis Bergquist* sp.	fascaplysin (**28**)	Red bis-indole alkaloid	Commercial	NCI-H417, DMS153, NCI-H526 (SCLC), H1299, A549, H23 (NSCLC), SCLC26A, GLC14, GLC16, and GLC19 (derived from SCLC patients)	134–1740 nM (96 h)	✗	Affects topoisomerase I, integrity of DNA, generation of ROS, apoptosis, and cell cycle arrest.	[31]
*Fascaplysinopsis Bergquist* sp.	fascaplysin (**28**)	Red bis-indole alkaloid	Commercial	NCI-H526, DMS53, DMS153, H69 (SCLC), A549, H1299, PC9 (NSCLC), SCLC26A and S457 (derived from pleural effusions of SCLC patients), IVIC-A, BH295 (derived from pleural effusions of NSCLC patients), and SCLC CTCs	0.53 ± 0.06, 1.17 ± 0.02, 1.35 ± 0.23, 1.05 ± 0.14, 0.63 ± 0.11, 0.69 ± 0.12, 0.99 ± 0.39, 1.48 ± 0.12, 0.2 ± 0.03, 1.41 ± 0.33, and 2.04 ± 0.05 IC_50_ (μM) NCI-H526, DMS53, DMS153, H69, A549, H1299, PC9, SCLC26A, S457, IVIC-A, and BH295, respectively	✗	ATM-triggered signaling cascade provoked by drug-induced DNA damage. Cytotoxic effect against lung cancer cell lines and spheroids of SCLC CTCs.	[37]
*Spongia* sp.	Langconol A (**29**); langconol B (**30**); langconol C (**31**); langcoquinone C (**32**); polyfibrospongol A (**33**); smenospongorine (**34**); langcoquinone A (**35**); langcoquinone B (**36**); dictyoceratin A (**37**); 19-hydroxypolyfibrospongol B (**38**); polyfibrospongol B (**39**); smenospongidine (**40**); nakijiquinone L (**41**); ilimaquinone (**42**); and smenospongine (**43**)	Merosesquiterpenes	Isolation	A549 (NSCLC) and WI-38 (NT)	**29**, **30**, **33**, **37**, and **39**: >50 and >50 **31**: 7.8 and 8.7 **32**: 6.6 and 8.2 **34**: 6.7 and 3.5 **35**: 9.9 and 8.4 **36**: 6.2 and 8.8 **38**: >50 and 42.1 **40**: 4 and 3 **41**: 8.9 and 6.9 **42**: 5.9 and 9.7 **43**: 7.8 and 9.2 IC_50_ (μM) A549 and WI-38, respectively (72 h)	✗	NR	[38]
*Spongia* sp.	Langcoquinone D (**44**), langcoquinone E (**45**), and langcoquinone F (**46**)	Sesquiterpenoid quinones	Isolation	A549 (NSCLC) and WI-38 (NT)	**44**: 8.9 and 5.6 **45** and **46**: >50 and >50 IC_50_ (μM) A549 and WI-38, respectively (24 h)	✗	NR	[39]
*Hyrtios erecta*	BA6 (heteronemin) (**47**)	Sesterterpen	Isolation	A549 (NSCLC)	5.12 μM (24 h)	✗	Induction of apoptosis by increasing mtROS and mitochondrial dysfunction. Bax upregulation; Bcl-2 downregulation; release of cytochrome C; activation of caspase-9 and -3.	[40]
*Ircinia ramose and Psammocinia*	irciniastatin A (**48**), also known as psymberin	Pederin family or pederin-type natural product	Chemical synthesis	A549 (NSCLC)	NR	✗	Translation inhibition that induces activation of ERK pathway and promotes the ectodomain shedding of TNF receptor 1.	[43]
*Smenospongia aurea*	smenamide A (**49**) and smenamide B (**50**)	Hybrid peptide/polyketide	Isolation	Calu-1 (NSCLC)	**49**: 48 nM **50**: 49 nM (72 h)	✗	Induction of apoptosis	[44]
*Dactylospongia* sp.	19-O-methylpelorol (**51**)	Sesquiterpene hydroquinone	Isolation	PC-9 (NSCLC)	9.2 μM (72 h)	✗	NR	[45]

* in vivo in lung cancer; not reported, NR; non-small-cell lung cancer, NSCLC; small-cell lung cancer, SCLC; non-tumoral, NT; reactive oxygen species, ROS; serine protein kinase, ATM; circulating tumor cell, CTC; mitochondrial ROS, mtROS; BCL2-associated X, Bax; B-cell lymphoma 2, Bcl-2; extracellular signal-regulated kinase, ERK; tumor necrosis factor, TNF; no in vivo evaluation in lung cancer, ✗.

### 2.3. Minority Orders

**Figure 5:**
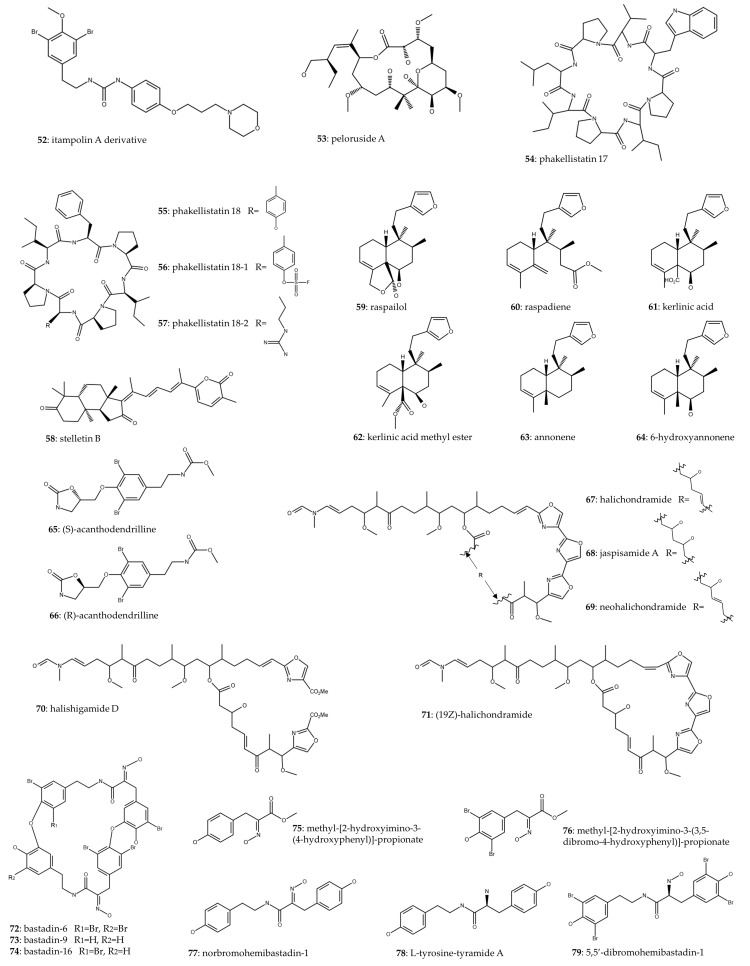
Chemical structure of compounds from minority orders.

Compounds included in this systematic review belonging to marine sponges of minority orders have been grouped in Table 3 and Figure 5. A team of researchers undertook the chemical synthesis of forty-five derivatives of itapolin A, a secondary metabolite identified in the sponge *Iotrochota purpurea*. From the array of derivatives, they selected the one itapolin A derivative (**52**) that exhibited the highest inhibition of p38α activity and evaluated its cytotoxic effects on the A549 cell line, revealing an IC_50_ of 0.66 μM [48]. Peloruside A (**53**) has been discovered within the marine sponge *Mycale henscheli*. The in vivo inhibitory effects on proliferation were compared with the standard anticancer drugs paclitaxel and docetaxel, and tumor growth inhibition (TGI) values improved those of the taxanes in the H460 and A549 models. In H460 xenografts, peloruside A (**53**) induced a dose-dependent reduction in tumor growth, with TGI values reaching 88% at 5 mg/kg and 99% at 10 mg/kg, resulting in no deaths. In A549 xenografts, TGI ranged from 51% to 74. Furthermore, the authors determined that the maximum tolerated dose of peloruside A (**53**) in mice ranged between 15 and 20 mg/kg, with mortality reaching 100% at the 20 mg/kg dosage after 8 days of treatment [49]. Otherwise, phakellistatins 17 (**54**) and 18 (**55**), two natural compounds derived from *Phakellia fusca*, were chemically synthesized alongside two analogs (**56**–**57**) of phakellistatin 18. The objective of synthesizing these analogs was to enhance the cytotoxic impact of phakellistatin 18 (**55**), which originally exhibited an IC_50_ of 72.42 μM. Among the synthesized analogs, only phakellistatin 18 analog 1 (**56**) demonstrated a slight improvement in cytotoxicity, yielding an IC_50_ of 67.53 μM [50].

**Figure 6:**
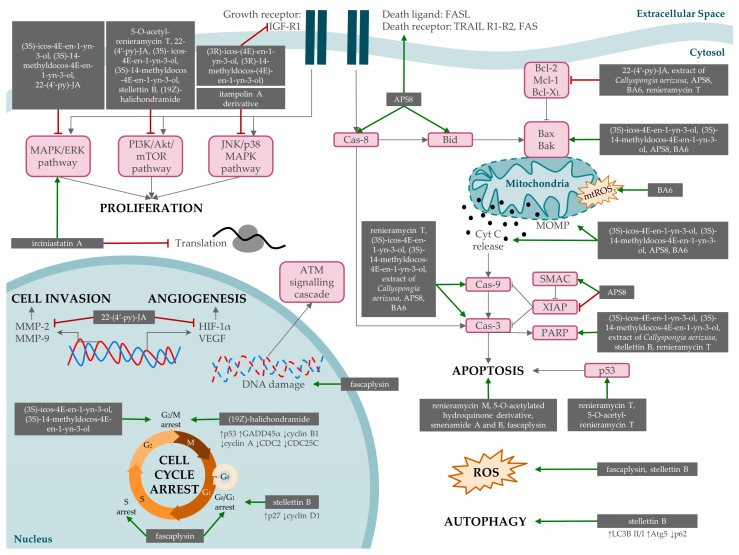
Mechanisms of action by which compounds derived from marine sponges exert their effect on lung cancer lines. Most of the mechanisms are related to proliferation, cell invasion, angiogenesis, cell cycle arrest, apoptosis, ROS production, and autophagy. Phosphatidylinositol 3-kinase, PI3K; protein kinase B, Akt; serine/threonine-protein kinase, mTOR; mitogen-activated protein kinase, MAPK; extracellular signal-regulated kinase, ERK; c-Jun N-terminal kinase, JNK; insulin-like growth factor 1 receptor, IGF-R1; Fas cell surface death receptor, FAS; Fas ligand, FASL; TNF-related apoptosis-inducing ligand, TRAIL; matrix metalloproteinase, MMP; hypoxia-inducible factor 1-alpha, HIF-1α; vascular endothelial growth factor A, VEGFA; tumor protein P53, p53; growth arrest and DNA damage inducible alpha, GADD45α; cyclin-dependent kinase-1, CDC2; cell division cycle 25C, CDC25C; cyclin-dependent kinase inhibitor 1B, p27; caspase, cas; BH3 interacting-domain death agonist, Bid; B-cell lymphoma 2, Bcl-2; induced myeloid leukemia cell-differentiation protein Mcl, Mcl-1; B-cell lymphoma—extra large, Bcl-XL; BCL2-associated X, Bax; Bcl-2 homologous antagonist/killer, Bak; reactive oxygen species, ROS; mitochondrial ROS, mtROS; cytochrome C, Cyt C; mitochondrial outer membrane permeabilization, MOMP; second mitochondria-derived activator of caspase, SMAC; X-linked inhibitor of apoptosis protein, XIAP; poly (ADP-ribose) polymerase, PARP.

In the study by Wang et al. (2016), stelletin B (**58**) was isolated from the marine sponge *Jaspis stellifera*, and its cytotoxic effect on A549 cells was demonstrated with an IC_50_ of 0.022 μM. The antitumor potential was exerted by targeting the PI3K/Akt/mTOR pathway. The findings indicated that the compound led to (1) G1 arrest, attributed to a reduction in cyclin D1 and increased p27 expression; (2) induction of apoptosis, associated with an increase in ROS generation and related to PARP cleavage; and (3) induction of autophagy [51]. The latter pathway of death is triggered by other drugs, such as metformin [52] or rapamycin [53]. This mechanism of action, as well as others previously mentioned, such as apoptosis or ROS induction, are described in Figure 6. Lhullier et al. (2019) isolated six clerodane diterpenes (**59**–**64**) from the marine sponge *Raspailia bouryesnaultae*. The compounds displaying the most potent cytotoxic activity were those containing a hydroxyl group at C-6, with IC_50_ values lower than 25 μM [54].

Acanthodendrilline was isolated from *Acanthodendrilla* sp., and its enantiomers were prepared from commercial materials. Cytotoxicity assessments demonstrated that (S)-acanthodendrilline (**65**) displayed approximately threefold greater potency against the H292 NSCLC cell line compared to (R)-acanthodendrilline (**66**) [55]. Five oxazole-containing macrolides (**67**–**71**) were isolated from the marine sponge *Chondrosia corticata*, with the most potent cytotoxic effect observed in (19Z)-halichondramide (**71**), exhibiting an IC_50_ value of 0.024 μM. Subsequent investigations into the mechanism of action of this compound revealed its capacity to induce a cell cycle arrest in the G2/M phase. Furthermore, it was found to suppress the Akt/mTOR signaling pathway and inhibit MAPK pathways, particularly ERK1/2 and p38 [56].

Mathieu et al. (2013) isolated three bastadins (**72**–**74**) from *Ianthella basta* and synthesized five chemical derivatives (**75**–**79**). Despite the absence of enhanced cytotoxic effects among the derivatives, 5,5′-dibromohemibastadin-1 (DBHB) (**79**) exhibited both in vitro growth inhibitory activity in cancer cells and anti-angiogenic properties. An in vivo assay conducted on B16F10 melanoma-bearing mice, which developed lung pseudometastases, revealed that the treatment with DBHB (**79**) did not confer significant therapeutic benefits in vivo. The authors suggest that another route of administration could improve outcomes [57]. Finally, a total of twenty derivatives of marine sponge alkaloids were synthesized, and their antiproliferative efficacy was assessed, revealing IC_50_ ranging from 14.15 to 43.72 μM for A549. The mechanism of action was investigated using the myeloid leukemia cell line HL-60, wherein cell cycle arrest, apoptosis, and depolarization of the mitochondrial membrane potential were observed. In an A549 xenograft model, the tested compound demonstrated a notable reduction in tumor growth to 354 ± 15 mm^3^ compared to untreated mice with 553 ± 66 mm^3^ [58].

Regarding the minor orders, the antitumor activity of macrolide derivatives and terpenes should be highlighted. In the first case, low IC_50_ values were obtained in the A549 tumor cell line, observing that certain derivatives showed more robust antitumor activity (compounds **67** and **71**) compared to the rest [56]. This highlights that small differences in molecular structure can lead to large changes in bioactivity. Similar macrolides have also been shown in previous studies to have synergistic activity with lansoprazole in lung cancer, causing lysosomal membrane permeabilization and leading to cell death in A549 and CAL27 cell lines [59]. Regarding stellettin B (**58**), it is a triterpene with high cytotoxic activity against A549 through the PI3K/Akt/mTOR pathway [51]. Other authors have reported that this compound possessed antitumor activity through the same molecular pathway in the glioblastoma cell line SF295 [60]. Furthermore, it should be noted that triterpenoids are one of the most abundant secondary metabolites in marine organisms and have previously been tested with high preclinical efficacy in animal models [61]. Finally, peloruside A (a polyketide, compound **53**) and alkaloid derivatives with a 2-amino-1H-imidazole core were tested in xenograft models from the H460 and A549 tumor lines, respectively, showing an ability to inhibit tumor growth without any remarkable systemic toxicity [49,58].

**Table 3:** Antitumor activity of compounds from minority orders in lung cancer.

Sponge	Compounds	Chemical Class	Methods of Production	Cell Line (NSCLC/SCLC)	IC50	In Vivo *	Molecular Mechanism	Ref.
*Iotrochota purpurea*	itampolin A derivative (**52**)	Brominated tyrosine alkaloid	Chemical synthesis	A549 (NSCLC)	0.66 μM (48 h)	✗	Decreased expression of phospho-p38	[48]
*Mycale henscheli*	peloruside A (**53**)	Polyketide	Isolation and chemical synthesis (both methods)	NR	NR	✓	(H460 xenografts) Dose-dependent decrease in tumor growth, with TGI values of 88% at 5 mg/kg and 99% at 10 mg/kg, with no deaths. (A549 Xenografts) TGI ranging from 51% to 74%	[49]
*Phakellia fusca*	phakellistatin 17 (**54**), phakellistatin 18 (**55**), phakellistatin 18 analog 1 (**56**), and phakellistatin 18 analog 2 (**57**)	Proline-rich cyclopeptides	Chemical synthesis	A549 (NSCLC)	**54**: >100 μM **55**: 72.42 μM **56**: 67.53 μM **57**: 79.71 μM (72 h)	✗	NR	[50]
*Jaspis stellifera*	stellettin B (Stel B) (**58**)	Isomalabaricane triterpene	Isolation	A549 (NSCLC)	0.022 μM (48 h)	✗	Targeting PI3K/Akt/mTOR pathway. Induction of G1 arrest (↑p27 and ↓cyclin D1), apoptosis (↑PARP cleavage and ↑ROS generation), and autophagy (↑LC3B II/I, ↑Atg5, and ↓p62).	[51]
*Raspailia bouryesnaultae*	raspailol (**59**), raspadiene (**60**), kerlinic acid (**61**), kerlinic acid methyl ester (**62**), annonene (**63**), and 6-hydroxyannonene (**64**)	Clerodane diterpenes	Isolation	A549 (NSCLC)	**59**: 24.12 μM **60**: 100.3 μM **61**: 66.22 μM **62**: 20.63 μM **63**: 143.7 μM **64**: 24.52 μM (48 h)	✗	NR	[54]
*Acanthodendrilla* sp.	(S)- acanthodendrilline (**65**) and (R)- acanthodendrilline (**66**)	Bromotyrosine alkaloid	Chemical synthesis	H292 (NSCLC)	**65**: 58.5 ± 6.7 μM **66**: 173.5 ± 24.7 μM (72 h)	✗	NR	[55]
*Chondrosia corticata*	halichondramide (**67**), jaspisamide A (**68**), neohalichondramide (**69**), halishigamide D (**70**), and (19Z)-halichondramide (**71**)	Oxazole-containing macrolide	Isolation	A549 (NSCLC)	**67**: 0.045 μM **68**: 32.63 μM **69**: 3.73 μM **70**: 1.65 μM **71**: 0.024 μM (72 h)	✗	G2/M cell cycle arrest (↑p53, ↑GADD45α, ↓cyclin B1, ↓cyclin A, ↓CDC2, and ↓CDC25C) and suppression of the Akt/mTOR signaling pathway (**71**).	[56]
*Ianthella basta*	bastadin-6 (**72**), bastadin-9 (**73**), bastadin-16 (**74**), methyl-[2-hydroxyimino-3-(4-hydroxyphenyl)]-propionate (**75**), methyl-[2-hydroxyimino-3-(3,5-dibromo-4-hydroxyphenyl)]-propionate (**76**), norbromohemibastadin-1 (**77**), L-tyrosine-tyramide A (**78**), and 5,5′-dibromohemibastadin-1 (**79**)	Bastadins	**72**–**74**: Isolation **4**–**8**: Chemical synthesis	A549 (NSCLC)	**72**: 3 μM **73**: 7 μM **74**: 8 μM **75**–**78**: >100 μM **79**: 68 μM (72 h)	✗	NR	[57]
NR	alkaloid analogs with 2-amino-1H-imidazole core	Alkaloid	Chemical synthesis	A549 (NSCLC)	14.15–43.72 μM (72 h)	✓	Inhibition of tumor growth in A549 xenograft models.	[58]

* in vivo in lung cancer; not reported, NR; tumor growth inhibition, TGI; non-small-cell lung cancer, NSCLC; non-tumoral, NT; phosphatidylinositol 3-kinase, PI3K; protein kinase B, Akt; serine/threonine-protein kinase mTOR, mTOR; cyclin-dependent kinase inhibitor 1B, p27; poly (ADP-ribose) polymerase, PARP; reactive oxygen species, ROS; microtubule-associated proteins 1A/1B light chain 3B, LC3B; autophagy-related 5, Atg5; tumor protein P53, p53; growth arrest and DNA damage inducible alpha, GADD45α; cyclin-dependent kinase-1, CDC2; cell division cycle 25C, CDC25C; no in vivo evaluation in lung cancer, ✗; in vivo evaluation in lung cancer, ✓; increase, ↑; decrease, ↓.

## 3. Materials and Methods

### 3.1. Study Eligibility

This systematic review attempts to aggregate the latest discoveries, specifically within the last 10 years, pertaining to bioactive molecules derived from marine sponges that exhibit antitumor activity in the context of lung cancer. This systematic review was undertaken following the guidelines outlined in the Preferred Reporting Items for Systematic Reviews and Meta-Analyses (PRISMA) 2020 statement [62] and was registered in the Open Science Framework under the DOI 10.17605/OSF.IO/CDN7U.

### 3.2. Inclusion and Exclusion Criteria

#### 3.2.1. Inclusion Criteria

The research articles included in this systematic review include those published between January 2013 and November 2023, featuring the utilization of extracts or compounds derived from marine sponges as well as chemically synthesized compounds identified in marine sponges and chemical analogs that have demonstrated antitumor activity against lung cancer.

#### 3.2.2. Exclusion Criteria

The research articles excluded in this systematic review include those in which the type of cancer analyzed was different from lung cancer, or the studied compound did not have its natural origin in a marine sponge, such as symbiotic fungi. Furthermore, research articles published in a language other than English and not available in full text were excluded. Finally, non-original articles, including reviews, systematic reviews, clinical cases, clinical trials, conference papers, letters, patents, book chapters, and notes, were also excluded.

### 3.3. Data Sources and Search Strategy

This systematic review was carried out using PubMed, Scopus, and Web of Science electronic databases to explore the bioactive molecules from marine sponges or synthetic analogs with antitumor effects against lung cancer. The research articles were obtained based on the search equations shown in Table 4.

### 3.4. Study Selection

Two of the authors (A.O.-P. and F.Q.) performed the bibliographic search in the three online databases and performed the screening procedure. Duplicates were eliminated, and research articles were selected by three steps of screening. The first screening was based on the titles and abstracts. A second screening was carried out by full-text reading of the publications that passed the first screening process. For the assessment of the articles in both screening steps, the inclusion and exclusion criteria previously established were taken into account. Finally, a third screening was conducted using a quality test that allowed assessing each of the selected articles. In the quality test, basic characteristics that a publication should have to quantify the antitumor effect of a molecule were evaluated, along with issues related to methodology, results, discussion, and proper statistical analysis. The articles were classified based on their score: low quality (0–11), medium quality (12–16), and high quality (17–20). Articles that scored less than 12 were excluded from this systematic review. All steps of the screening process were carried out by two of the authors (A.O-P and F.Q). In case of disagreement, a discussion was conducted until a consensus was reached. Otherwise, the intervention of a third author was required to make a final decision.

### 3.5. Data Extraction

Once the list of articles included in this study was obtained, data were extracted and classified independently by the same authors. A table was generated, including data related to the marine sponge (species), studied bioactive compound (compound name, chemical class, and method of production), cytotoxic effect (IC_50_ in studied cell lines and classification into SCLC or NSCLC), in vivo assays, and the molecular mechanism of action. Missing data were noted as ‘not reported (NR)’. Studies were grouped according to the orders of the analyzed marine sponge.

## 4. Limitations and Future Perspectives

Lung cancer exhibits elevated mortality, incidence, and prevalence rates, so the exploration and development of novel drugs and therapeutic alternatives are mandatory. Previous studies have generated much interest in the identification of new molecules with a wide range of antitumor activities. In fact, alkaloids such as renieramycin M (**1**), derivatives of jorunnamycin A (**4**) and hydroquinone 5-O-monoester (**2**), peptide/polyketide hybrids such as smenamide A (**49**) and smenamide B (**50**), macrolides (compounds **67** and **71**), and finally triterpenes such as stellettin B (**58**) have been shown to be the most effective antitumor compounds. In this context, the review aims to be a tool for researchers working in this field to learn about the main molecules that show relevant efficacy in lung cancer, including those with low incidence. Knowledge of these natural compounds and their derivatives will be a source of inspiration for their structural modification and for continuing to analyze different marine species. These studies will allow us to select the most promising molecules to carry out clinical research into new treatments for patients with lung cancer, improving their quality of life and prognosis.

To date, there are two compounds isolated from marine sponges approved by the FDA for the treatment of various cancers. One is cytarabine, Cytosar-U^®^, approved in 1969 and developed by Pfizer for the treatment of leukemia. The other is Eribulin Mesylate, Halaven^®^, approved in 2010, produced by Eisai Inc. and indicated for the treatment of metastatic breast cancer and metastatic liposarcoma [63]. Furthermore, none of the compounds included in this systematic review are currently in clinical trials. Nevertheless, various compounds derived from marine sponges that exhibit potent antitumor properties are currently under development in different phases of clinical trials in oncology. Recently, antibody–drug conjugates (ADCs) have gained attention for the development of antitumor drugs. These compounds preserve the potent cytotoxic properties of natural substances with the precise targeting of antibodies, achieving the elimination of tumor cells in an effective and precise manner [64]. In fact, two of the three marine sponge-derived drugs currently in clinical trials are ADCs. STRO-002 is an ADC that contains taltobulin, a synthetic analog derived from hemiasterlin, originally sourced from the marine sponge *Hemiasterella minor*. Phase I clinical trial for STRO-002 is in progress in ovarian cancer and endometrial cancer patients (NCT03748186, NCT05200364, and NCT05870748) [65]. E7130, a novel antitumor agent inspired by the natural compound norhalichondrin B [66], is currently undergoing evaluation in phase I clinical trials of solid tumors (NCT03444701). Finally, farletuzumab ecteribulin (MORAb-202), an ADC comprising farletuzumab linked to eribulin mesylate [67], is currently in phase II clinical trials for the treatment of NSCLC (NCT05577715), ovarian carcinoma (NCT05613088), and solid tumors (NCT04300556).

However, there are some advantages and disadvantages associated with these sponge-derived compounds. The low number of sponge-derived products, both FDA-approved and in clinical trials, is remarkable despite an intense search that has yielded 11,000 natural sponge-derived products. Despite promising in vitro results in therapeutic trials, many lack efficacy, show toxicity, are unstable, or exhibit poor pharmacokinetics when tested in in vivo models [68]. The toxicity is a key factor for the success of antitumor therapies, and the reduction of side effects caused by the treatment depends on it. On the one hand, regarding in vitro assays, most of the included studies do not test the compounds in vitro in non-tumor cell lines, which is a major limitation in elucidating their potential toxicity. However, some studies show a high difference in IC_50_ values between tumor (more sensitive) and non-tumor lines, which may indicate that these bioactive molecules may possess treatment specificity for tumor cells. This occurs in the MRC-5 cell line with compounds **16** (a 3-al-kylpyridinium analog) and **26** (an amino derivative) treatments [25,30]. Conversely, other compounds such as (−)-petrosynoic acids (**17**–**20**) and pellynols (**21**–**25**) [26] or merosesquiterpenes (**29**–**43**) [38] showed no differences in the cytotoxic effect even a lower IC_50_ in non-tumor lines compared to tumor cell lines. On the other hand, among the publications that include in vivo tests, only one of them includes data on the toxicity of peloruside A (**53**), indicating the maximum dose tolerated by mice [49], and other authors determine the pharmacokinetic parameters of 22-(4′-py)-JA (**4**) [17].

Of the 33 articles included in this systematic review, only 5 analyzed the in vivo effects of these compounds in different murine models [17,29,49,57,58]. In fact, approximately 95% of recently developed anticancer drugs ultimately prove unsuccessful in clinical trials despite exhibiting robust anticancer effects in established in vitro preclinical models [69]. A possible approach to overcome this limitation is to load these compounds into drug delivery systems (DDS). DDSs facilitate the controlled drug release regarding dosage, site-specific targeting, and duration within the organism. Through modulation of drug absorption, distribution, metabolism, and excretion processes, DDSs contribute to the increase in therapeutic efficacy and safety profiles of natural compounds. Delivery systems include micro/nanostructures, biopolymers, and antibody–drug conjugates [68]. For instance, avarol, a water-insoluble compound from the marine sponge *Dysidea avara*, was integrated into a liposomal formulation to overcome this problem [70]. Furthermore, the potential of using marine sponge-derived metabolites lies in the opportunity to modify the skeleton or active core of each compound, using it as a vehicle for the development of new derivatives that improve the cytotoxic effect and reduce the side effects of the original molecule [71].

In addition to their potential toxicity, another issue that needs to be addressed is difficulties in obtaining sponges from their natural environment and the availability of these sponge-derived secondary metabolites, which are frequently found in trace amounts. In addition, the cost of production must be affordable. So, if they show potential as antitumor agents, chemical synthesis or other pathways of obtaining should be developed so that they can be produced on a large scale [72].

Finally, this systematic review shows some methodological limitations: (1) the publications before 2013 were excluded from analysis; (2) the term “marine sponge” in the search equation limits the possibility of finding products found naturally in sponges but which have been chemically synthesized if the authors have not included this term in their publications; and (3) obtaining a robust conclusion is challenging due to the limited amount of data available to each of the compounds and the heterogeneity of the data; therefore, caution should be applied when interpreting the obtained results.

## 5. Conclusions

The aim of this systematic review was to analyze the current state-of-the-art in the discovery of new antitumor drugs derived from marine sponges and applied to lung cancer. Lung cancer is a widespread health problem worldwide and needs new therapeutic strategies to reduce mortality. In addition, research focuses on large-cell lung cancer, with small-cell lung cancer being less frequently studied. Natural compounds, and, more specifically, those derived from or inspired by marine sponges, are a valuable source of inspiration for developing new drugs. However, although much research is being carried out in this field, further analysis is needed before reaching the clinic, including in vivo assays and clinical trials. In this way, we provided a broad overview of how current research in this field is progressing.

## Figures and Tables

**Table 4:** Search equations in each database.

Database	Search Strategy
PubMed	((Lung Neoplasms[MeSH Terms]) OR (Lung Cancer[Title/Abstract]) OR (Pulmonary Cancer[Title/Abstract]) OR (Pulmonary Neoplasms[Title/Abstract])) AND ((porifera[MeSH Terms]) OR (Demospongiae[Title/Abstract]) OR (marine sponge[Title/Abstract])) AND ((Antineoplastic Agents[MeSH Terms]) OR (Bioactive*[Title/Abstract]) OR (Antitumor*[Title/Abstract]) OR (Anticancer*[Title/Abstract]))
Scopus	(TITLE-ABS (lung AND neoplasms) OR TITLE-ABS (lung AND cancer) OR TITLE-ABS (lung AND adenocarcinoma) OR TITLE-ABS (pulmonary AND cancer) OR TITLE-ABS (pulmonary AND neoplasms)) AND (TITLE-ABS (porifera) OR TITLE-ABS (demospongiae) OR TITLE-ABS (marine AND sponge)) AND (TITLE-ABS (bioactive) OR TITLE-ABS (antitumor) OR TITLE-ABS (anticancer) OR TITLE-ABS (antineoplastic))
Web of Science	((TI = (Lung Neoplasms OR Lung Cancer OR Pulmonary Cancer OR Pulmonary Neoplasms)) OR (AB = (Lung Neoplasms OR Lung Cancer OR Pulmonary Cancer OR Pulmonary Neoplasms))) AND ((TI = (porifera OR demospongiae OR marine sponge)) OR (AB = (porifera OR demospongiae OR marine sponge))) AND ((TI = (bioactive OR antitumor OR anticancer OR antineoplastic)) OR (AB = (bioactive OR antitumor OR anticancer OR antineoplastic)))

## Data Availability

The data presented in this study are available in the main article.
